# Boosting regulatory T cell function for the treatment of autoimmune diseases – That’s only half the battle!

**DOI:** 10.3389/fimmu.2022.973813

**Published:** 2022-08-10

**Authors:** Janine Schlöder, Fatemeh Shahneh, Franz-Joseph Schneider, Björn Wieschendorf

**Affiliations:** ^1^ Department of Dermatology, University Medical Center of the Johannes Gutenberg-University, Mainz, Germany; ^2^ ActiTrexx GmbH, University Medical Center of the Johannes Gutenberg-University, Mainz, Germany

**Keywords:** cellular therapy, regulatory T cell, T effector cell, tolerance, autoimmunity, immune regulation

## Abstract

Regulatory T cells (Treg) represent a subset of specialized T cells that are essential for the regulation of immune responses and maintenance of peripheral tolerance. Once activated, Treg exert powerful immunosuppressive properties, for example by inhibiting T cell-mediated immune responses against self-antigens, thereby protecting our body from autoimmunity. Autoimmune diseases such as multiple sclerosis, rheumatoid arthritis or systemic lupus erythematosus, exhibit an immunological imbalance mainly characterized by a reduced frequency and impaired function of Treg. In addition, there has been increasing evidence that – besides Treg dysfunction – immunoregulatory mechanisms fail to control autoreactive T cells due to a reduced responsiveness of T effector cells (Teff) for the suppressive properties of Treg, a process termed Treg resistance. In order to efficiently treat autoimmune diseases and thus fully induce immunological tolerance, a combined therapy aimed at both enhancing Treg function and restoring Teff responsiveness could most likely be beneficial. This review provides an overview of immunomodulating drugs that are currently used to treat various autoimmune diseases in the clinic and have been shown to increase Treg frequency as well as Teff sensitivity to Treg-mediated suppression. Furthermore, we discuss strategies on how to boost Treg activity and function, and their potential use in the treatment of autoimmunity. Finally, we present a humanized mouse model for the preclinical testing of Treg-activating substances *in vivo*.

## Introduction

Regulatory T cells (Treg) are important mediators of peripheral tolerance and maintenance of immunological homeostasis (1). In autoimmune diseases such as multiple sclerosis (MS), rheumatoid arthritis (RA) or systemic lupus erythematosus (SLE), the function and frequency of Treg are severely impaired (2–5). To counteract this dysfunction, adoptive transfer of Treg as well as the potential use of Treg-enhancing substances represent attractive therapeutic options for the treatment of immune-mediated pathologies such as autoimmune diseases (6–8). In 2018, the effectiveness of adoptively transferred autologous Treg was tested in one SLE patient for the first time, in a study by Dall’Era et al. (9). Their results suggest that this treatment leads to an increased amount of activated Treg in the inflamed skin of the patient. However, a Treg-based therapy can only work efficiently if the autoreactive T effector cells (Teff) are also responsive to the suppressive function of Treg. In the context of MS, RA, and SLE, a significantly reduced ability to suppress Teff in the peripheral blood of patients has been observed over the past decade (10–12). This mechanism of decreased T cell responsiveness to Treg-mediated suppression is termed Treg resistance. These findings suggest that strategies for treating autoimmune diseases probably need to be optimized. Since there is a defect on both sides – Treg and Teff -, it could be beneficial to address both deficiencies in one therapeutic approach. In this review, we present the problem of Treg dysfunction and Treg resistance in patients with autoimmune diseases. We summarize how certain immunomodulating therapies in the treatment of autoimmune disease affect Treg function and frequency, and the responsiveness of T cells for Treg-mediated suppression. Furthermore, we discuss potential therapies to specifically boost Treg function, as well as several *in vitro* and *in vivo* methods for the preclinical testing of such Treg-activating substances.

## Characterization and subpopulations of human Treg

Treg are a distinct heterogeneous population of CD4^+^ T cells with high expression of forkhead box P3 (Foxp3) transcription factor which is associated with the suppressive function of this cell population (13, 14). Treg participate in controlling many physiological processes by maintaining immunological homeostasis and thus protecting our body from developing diseases, such as autoimmune diseases or cancer. Lack of Foxp3^+^ Treg or loss-of-function mutations of Foxp3 results in devastating autoimmunity in humans (15). Immune dysregulation, polyendocrinopathy, enteropathy, and X-linked syndrome (IPEX), an early and severe form of autoimmunity, represents an example of diseases directly associated with loss-of-function mutation of Foxp3 (16, 17). Therefore, deficiency in either the number or the function of Treg in the peripheral blood is a decisive factor in the regulatory burden on the immune system (18).

The majority of Treg repertoire in secondary lymphoid organs originates from the thymus (19). Treg lineage with a distinct T cell receptor (TCR) repertoire naturally develops from the autoreactive thymocytes in the thymus and forms a suppressive population specific to self-antigens, termed thymus-derived Treg (tTreg) (20, 21). During thymic development, T cells encounter high-affinity self-antigens presented by thymic epithelial cells and are clonally deleted through negative selection at the early stage of T cell development during central tolerance (22). Low to intermediate-affinity antigen recognition results in a positive selection of T cells which may leave the thymus and enter the periphery. Only a minority of T cells differentiate to Treg lineage at CD3^+^CD4^+^CD8^+^CD25^+^ stage in the thymus with an intermediate degree of binding to antigens (23). Signals mediated through interaction with TCR result in the upregulation of the high-affinity IL-2 receptor α -chain (CD25) on the cell surface of Treg, which is essential for the maintenance of Foxp3 expression. Following TCR stimulation, the IL-2 signaling cascade is the key second to TCR-mediated signaling in Treg development, lineage differentiation, and function (24). Studies have shown that IL-7 and IL-15 signaling may also play an important role in the survival and differentiation of Foxp3^+^ cells (25, 26).

Moreover, Treg can derive from naive conventional CD4^+^ T cells, which differentiate into Foxp3^+^ Treg in secondary lymphoid organs upon antigen exposure, cytokines stimulation, metabolic intermediates, and hormones in the periphery under both non-inflammatory and inflammatory conditions (27, 28). The development of peripheral Treg (pTreg) appears in the mucosal surfaces and the lamina propria of the colon and small intestine which confer immune tolerance to food- and microbiota-derived antigens (29). In addition to IL-2, transforming growth factor β (TGF-β), retinoic acids (RA), and short-chain fatty acids (SCFA) can induce Foxp3 expression in naïve T cells *in vitro*, however, TGF-β stimulation alone does not produce fully suppressive and stable Foxp3^+^ cells *in vitro* (30, 31).

Noticeably, tTreg and pTreg share significant features of Treg characteristics. In humans, substantial numbers of activated CD4^+^ T cells express low levels of Foxp3 which make it challenging to describe Treg based on Foxp3 positivity alone (32). Treg are also characterized by high expression of the cytokine receptor CD25 and are mostly dependent on IL-2 cytokine signaling while CD4^+^ T cells require IL-7 cytokine signaling (33). Therefore, in humans, as CD25 is expressed by recently activated CD4^+^ T cells, low levels of the IL-7 receptor α -chain (CD127) can be a suitable marker to identify human Treg as CD3^+^CD4^+^CD25^high^CD127^low^Foxp3^+^ cells (34). However, there is still no definitive marker for the characterization of human Treg. Additionally, Treg express distinct surface receptors to adjust in various peripheral functional profiles, defined as Treg subsets (35).

Previous studies determined that tTreg have a stable Treg phenotype in the periphery since the epigenetic stabilization of Foxp3 expression is also imprinted in the thymus. The Treg phenotype and function maintain stable by the DNA demethylation of conserved noncoding region 2 (CNS2) in the human Foxp3 locus, known as the Treg-specific-demethylated region (TSDR) in thymic-derived Treg. CNS2 is a TCR-responsive enhancer with binding sites for Runx1–CBFβ transcription-factor complexes (36–38). CNS2 is completely demethylated in tTreg but methylated in naïve or activated CD4^+^ Teff despite transient upregulation of Foxp3 (39), therefore they can be distinguished from each other. Several other transcription factors cooperate with Foxp3 to stabilize the expression of the Treg signature such as CTLA-4 (cytotoxic T lymphocyte antigen 4), GITR (glucocorticoid-induced TNF receptor), and ICOS (inducible T cell co−stimulator) (40, 41).

Naïve Treg (nTreg) are the majority of Treg in the peripheral blood and secondary lymphoid organs with strong suppressive capacity. This resting population is characterized as Foxp3^low^CD4^+^CD45RA^high^CD45RO^low^CD25^low^ cells (42). Effector Treg make up a small activated population of Treg in the circulation and lymphatics. Effector Treg share phenotypic features with activated CD4^+^ Teff as Foxp3^high^CD4^+^CD45RA^low^CD45RO^high^CD25^high^ (35, 42, 43). This Treg subpopulation displays a greater capacity to migrate into non-lymphoid or inflamed tissues to maintain immunological tolerance or adopt additional functions to modulate immune homeostasis. Current studies showed that HLA-DR (human leukocyte antigen DR), TIM-3 (T cell immunoglobulin mucin-3), and ICOS, which are mainly expressed by effector Treg, can be used as additional markers to characterize this Treg subset with suppressive function (44, 45). However, the nTreg population is not a completely homogeneous population and partially expresses these markers in some inflammatory contexts too. Thus, it is difficult to determine both of functional subsets – nTreg versus effector Treg - with specialized markers in complex milieus. Additionally, similar markers in a healthy individual may not necessarily express the same in the potentially abnormal Treg in autoimmune settings. Therefore, identifying better markers for characterizing human Treg may help us advance more detailed information on this important cell population.

## Treg function in steady state and disease

Treg control both immune and non-immune cell responses by different mechanisms to mediate peripheral tolerance in the steady-state. Until now, some functional mediators of Treg suppression have been recognized. Treg produce inhibitory cytokines such as IL-10, IL-35, and TGF-β to inhibit Teff activation in various immune responses (46–49). Treg interact with dendritic cells (DC) and modulate their maturation and/or function. CTLA-4 is a crucial molecule for Treg’s suppressive properties and is constitutively expressed on the cell surface of Treg. The CTLA-4 deficiency in the Treg population causes a shift in susceptibility to different autoimmune diseases. CTLA-4 can exclude Teff from contact with DC. CTLA-4 has a higher affinity to bind its costimulatory molecules CD80 (B7-1) and CD86 (B7-2) on DC, thereby interfering with the binding of CD28 in Teff, thus diminishing DC’s capacity to activate T cells (50, 51).

However, the expression of other immune checkpoint molecules such as lymphocyte activation gene-3 (LAG3) can partly reimburse for the loss of CTLA-4 and suppress DC maturation as well (52). Treg can also impede antigen-specific Teff and reduce the capacity of the DC to present antigen by removing MHC class II-antigen complexes from the surface of DC. Treg have a higher trogocytic capacity than T cells by uptake of DC membranes without the CTLA-4-mediated mechanisms (53).

Cytolysis by Treg is mediated by granzyme B and perforin in humans (54). Furthermore, Treg can induce the production of the immunoregulatory molecule indoleamine 2, 3-dioxygenase (IDO) in DC (55). Treg consume more IL-2 with the expression of the high-affinity α-chain receptor for IL-2 and deprive self-reactive Teff or natural killer cells, which express only the low-affinity IL-2 receptor complex (56). The consumption of available IL-2 by Treg also adjusts the proliferative or apoptotic rates of Treg to keep their population size stable (57).

Overproduction of IL-2 during an active immune response such as infection permits Treg numbers to follow with inflammation. Therefore, the excessive expansion of Treg in some acute inflammatory responses leads to an anergic phase and immune suppression (58, 59).

Besides extracellular factors, Treg also use intracellularly expressed factors to exercise their suppressive function. Several studies showed that Treg can disrupt the metabolic activity of conventional T cells through the transfer of the inhibitory second messenger cyclic adenosine monophosphate (cAMP) *via* gap junctions, the production of adenosine by CD39 and CD73, and the subsequent activation of the adenosine receptor 2A (A2A) on T cells (60–63).

Recent studies describe the functional role of Treg beyond their suppressive capacity, which populate in specific tissues (64). Effector Treg can adapt and gain additional functional properties by expressing unique homing receptors, transcription factors, immunomodulatory mediators, and TCR repertoire to maintain the steady-state homeostasis in the different local milieus (31, 65). They can be found in non-lymphoid tissues such as the skin, lungs, liver, gastrointestinal tract, adipose tissue, muscle, central nervous system, and placenta (66). The presence of tissue-resident Treg makes it possible to respond and expand faster after local damage or inflammation. Treg are able to adjust their molecular programs in distinct niches and gain unique effector functions tailored for local immune regulation, tissue homeostasis, and regenerative processes (64).

In the visceral adipose tissue (VAT), Treg are highly abundant and different from their counterparts in lymphoid tissues. VAT Treg show an increased IL-33 secretion as well as expression of C-C motif chemokine receptor 2 (CCR2) and CCR24. Furthermore, levels of specific transcription factors such as GATA-3 and the adipocyte regulator peroxisome proliferator-activated receptor−γ (PPARγ) are also elevated (67, 68). Moreover, VAT Treg maintain the balance between anti-inflammatory and pro-inflammatory macrophages, promoting the differentiation of the anti-inflammatory monocyte-macrophage population (31, 69). In the skeletal muscle, tissue-resident Treg increase after muscle injury with a unique TCR repertoire and produce the epidermal growth factor amphiregulin and ST2 (the receptor for the alarmin IL-33). Localized Treg in muscle tissue control inflammation after injury by modulating myeloid cells from a pro-inflammatory to a reparatory state and start regenerating muscle satellite cells to potentiate muscle repair (70, 71).

In the vascular system unlike other non-lymphoid tissue such as VAT or muscle, Treg are rare in steady-state but we have recently shown that Treg accumulate in the thrombus over time and form a resident Treg population. Clot Treg are a special subset, characterized by the expression of secreted protein acidic and rich in cysteines (SPARC) and secretion of IL-18, IL-33, and amphiregulin. Clot Treg boost matrix metalloproteinases (MMP) activity by monocytes and facilitates the resolution of thrombi in mice (72). However, we still need to know whether such a Treg population develops in humans upon induction of thrombosis and increases the resolution of thrombi. Taken together, the non-immune functions of Treg seem to be critical for tissue homeostasis and repair in response to different stimuli. However, how these additional functions are imprinted in Treg remains unknown.

Investigations on Treg function in VAT, muscle, and clot revealed that Treg may acquire additional unique functions to maintain homeostasis in tissues that are distinct from their canonical suppressive function (68, 71, 72). To what extent these findings can be related to other pathological settings, such as autoimmune disease, still remains unclear. Since tissue Treg have similar transcription factors to other tissue cells, it is possible that such a population can develop locally during the progression of an autoimmune disease. However, it is not clear whether these additional functional activities of tissue Treg have beneficial or detrimental impacts on the development and the pathogenesis of autoimmune diseases. We propose that the signals directing the differentiation of tissue Treg population may affect organ-specific tissue damages. Nevertheless, intensive scientific studies are necessary to clarify whether tissue Treg play an active role in the pathogenesis of other diseases.

Effector Treg sense the inflammatory milieu of conventional CD4^+^ T helper cell (Th) -mediated responses and polarize further by the expression markers related to Th-like phenotypes (73). This specific polarization confers Treg the ability to migrate to the site of a specific type of inflammation and restrain the corresponding T cells. Treg subsets expressing specific chemokine receptors and transcription factors are detectable in the peripheral blood of patients with different disease settings. Recent studies revealed that Treg subtypes in the peripheral blood showed effector-like properties of Th17, Th1, and Th2 cells (45, 74, 75).

Cytokines determine the quality of an effector T cell response and reprogramming of Treg. The expression of IFN-γ by Th1-like Treg requires STAT1 and T-bet (T-box expressed in T cells) expression (76, 77), IL-6-driven Th17-like Treg express RORγt and STAT3 for the secretion of IL-17A, and IL-4-driven Th2-like Treg upregulate IRF4 and GATA-3 (78). However, it is unclear whether Th-like Treg are differentiated before entering the inflammatory niche or whether they are locally adapted to a distinct inflammatory response.

**Figure 1:**
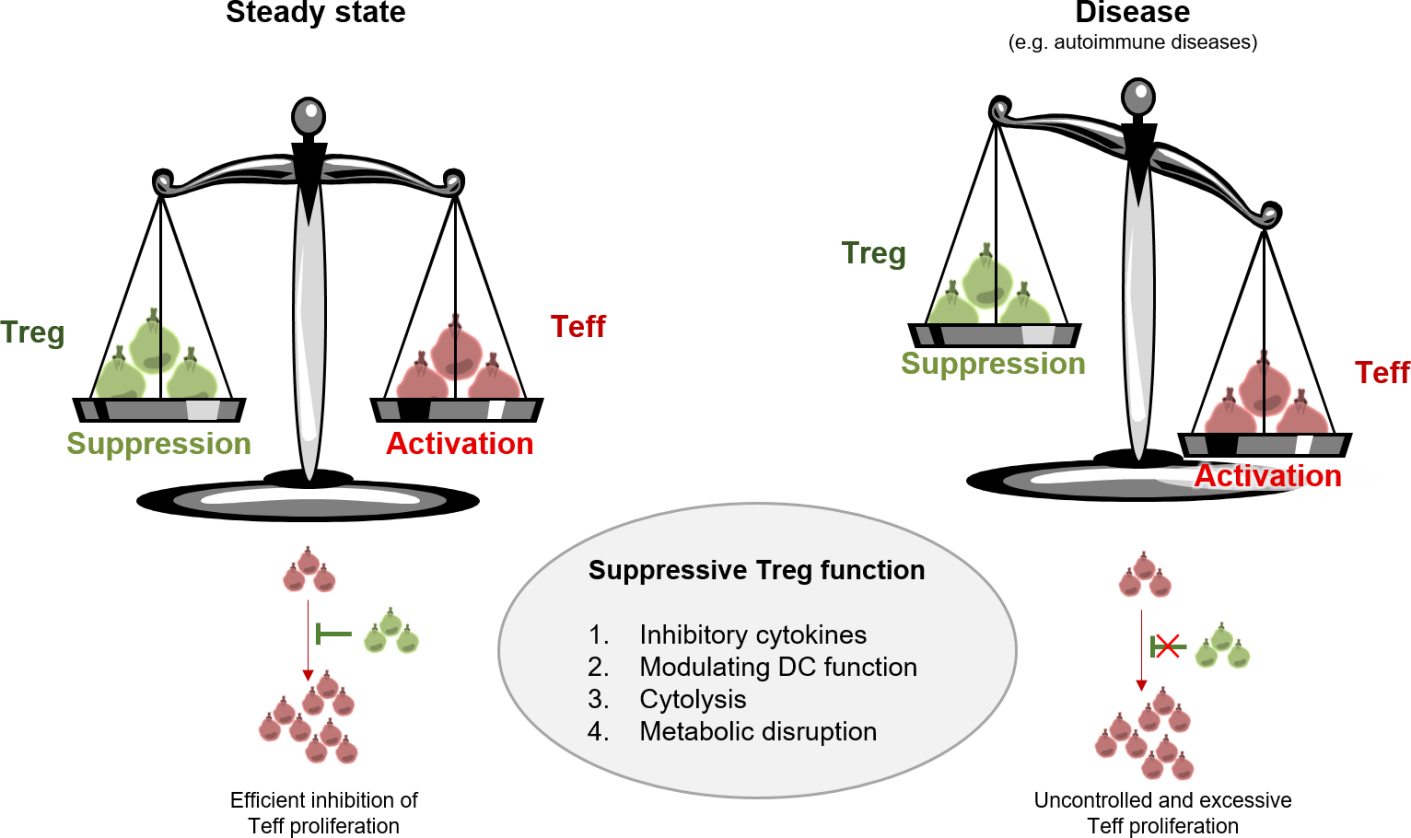
Treg function in steady state versus disease. Treg are active mediators of peripheral tolerance and protect us from excessive immune responses including autoimmunity. Treg maintain homeostasis mainly through four modes of action: 1. inhibitory cytokine production, 2. modulating DC function, 3. cytolysis, and 4. metabolic disruption. Aberrant Treg function results in disturbance of immune homeostasis and uncontrolled proliferation of Teff.

In patients with autoimmune diseases, such as type 1 diabetes (T1D) or MS, the frequency of IFN-γ^+^ Foxp3^+^ tTreg with lower suppressive activity is augmented in the blood compared to healthy donors. These Th1-like Treg upregulate the transcription factor T-bet and chemokine receptors like CCR5 and CXCR3 (79–81). It was shown that the frequency of Th2-like Treg is increased in the skin of patients with systemic sclerosis (SS). The enhancement of Th2-like Treg was accompanied by the secretion of IL-4 and IL-13 and upregulation of ST2, GATA-3, and IRF-4. However, it is not clear whether these Treg have a functional role in disease pathogenesis. While IPEX distinctly demonstrates the fundamental function of Treg, it was shown that defects in the genetic or environmental origin of Treg may drive the immune system towards pathology in the context of unwanted or insufficient immune responses. However, it is an emerging concept that Treg instability, defects in trafficking abilities, and very distinct heterogeneity imprinted by local niche might be important causes in the dysregulation of Treg function as well (**Figure 1**) (82).

It is known that changes of phenotype, function, and population size of Treg lead to autoimmunity and deployment of autoimmune diseases such as RA (83), T1D (84), and MS (80).

Studies showed that the significant reduction of Treg in the peripheral blood of RA patients may be associated with the initiation and progression of RA. However, the total Treg frequency was not significantly different between RA patients and healthy individuals rather CD25 expression was downregulated in the total Treg population (4). Findings on T1D patients displayed that the inhibitory function of Treg on the proliferation of autologous Teff was impaired mainly age-dependently by reduced production of IL-2, IFN-γ, and TGF-β (85, 86). In the case of MS, the numbers of recent thymic emigrant (RTE) naive Treg, characterized as CD4^+^CD25^+^CD45RA^+^CD45RO^−^Foxp3^+^CD31^+^, are significantly reduced within the peripheral blood of MS patients age-dependently. It has been suggested that the reduction of RTE Treg may be compensated with the higher expansion of effector Treg and may contribute to the Treg defect associated with MS (2). Additionally, studies by Venken et al. revealed that the suppressive function of nTreg is impaired in both early and chronic stages of MS disease and that most effector Treg will be expanded during the chronic stage of disease progression (3). Taken together, there is a large body of evidence that Treg play a pivotal role in a variety of autoimmune circumstances (**Table 1**) (80, 87–95). Apart from the alterations in the function and frequency of Treg in patients with autoimmune diseases, a number of recent studies also observed a disruption on the side of Teff. Such T cells exert a disturbing lack of sensitivity towards Treg-mediated suppression, a mechanism termed Treg resistance.

**Table 1:** Impaired Treg function in autoimmune diseases.

Autoimmune disease	Study
Autoimmune hepatitis	Longhi et al. (87)
Autoimmune polyglandular syndrome type II	Kriegel et al. (88)
Type 1 diabetes	Lindley et al. (89)
Hepatitis C-mixed cryoglobulinemia vasculitis	Boyer et al. (90)
Idiopathic thrombocytopenic purpura	Ling et al. (91)
Immunodysregulation polyendocrinopathy enteropathy X-linked	Bacchetta et al. (92)
Multiple sclerosis	Viglietta et al. (80)
Myasthenia gravis	Balandina et al. (93)
Sarcoidosis	Rappl et al. (94)
Systemic lupus erythematosus	Alvarado-Sánchez et al. (95)

This table provides an overview of individual autoimmune diseases with potential pathologic findings in Treg function, and the corresponding scientific study.

## Treg resistance in autoimmune diseases

Successful suppression of T cell activation depends not only on efficient Treg function, but also on T cell susceptibility to Treg-mediated suppression. T cells are crucial for triggering and mediating immune responses, for example as a protection against pathogens in the context of an infectious disease, but also in adverse reactions to self-antigens in the context of autoimmune diseases. T cell-mediated immune responses are generally controlled by Treg-based regulatory mechanisms (96). Under physiological conditions, T cells are able to transiently evade Treg-mediated control to initiate a protective response against an infectious agent. In recent years, however, there has been increasing evidence that T cells can overcome Treg-mediated suppression even under non-physiological conditions, thereby contributing to the pathogenesis of autoimmune diseases such as RA, T1D and MS (97–99). This mechanism is commonly referred to as Treg resistance. In 2013, Trinschek et al. and Schneider et al. reported for the first time about Treg resistance in T cells from relapsing-remitting MS (RRMS) patients. Thus, isolated CD4^+^CD25^-^ Teff from the peripheral blood of RRMS patients are characterized by a reduced sensitivity for Treg-mediated suppression (97).

But how can T cells overcome regulation by Treg? In recent years, some research work has focused on identifying molecular signaling pathways associated with Treg resistance in T cells. A broad spectrum of extracellular as well as intracellular molecules can affect T cell responsiveness towards Treg-mediated suppression (100). IL-6 is the most prominent cytokine that can induce Treg resistance in T cells. Studies have already confirmed that elevated IL-6 levels play a pathological role in MS and RA (101, 102). Isolated T cells from the peripheral blood of RRMS patients exhibit an accelerated IL-6 production, which correlates with a decreased suppression of T cell proliferation (10). In addition, Treg-resistant T cells are marked by an increased expression of IL-6 receptor (IL-6R). Direct blockade of IL-6R by an anti-IL-6R antibody leads to abolition of Treg resistance in T cells (10). Furthermore, binding of IL-6 to its specific receptor induces phosphorylation of the transcription factor STAT3 (signal transducer and activator of transcription 3). Interestingly, elevated levels of phosphorylated STAT3 molecules have been detected in T cells from the peripheral blood of RRMS patients. These findings correlate with the severity of the disease and impaired suppressibility of T cells (97).

IL-6-STAT3 signaling initiates the activation of protein kinase B (PKB/c-akt) in immune cells. Over the last years, there has been increasing evidence that T cell sensitivity towards Treg-mediated regulation depends on the activation state of PKB/c-akt (103). In mice, the targeted knockout of TRAF6 (TNFR-associated factor 6) and Cbl-b, both molecules that negatively regulate PKB/c-akt activation, allows T cells to evade immune suppression by Treg (104, 105). Further studies by Wehrens et al. revealed an increased phosphorylation of PKB/c-akt in T cells isolated from patients with juvenile idiopathic arthritis (106). This hyperactivation of PKB/c-akt correlates with a significantly reduced responsiveness of T cells to Treg-mediated suppression. Similar results were obtained in the context of MS: Treg-resistant T cells from the peripheral blood of RRMS patients are also characterized by an enhanced phosphorylation of PKB/c-akt (97). Specific inhibition of PKB/c-akt activation restores T cell responsiveness to Treg-mediated suppression (43). Thus, the activation and regulation of PKB/c-akt seems to be a central node in the development of Treg resistance in T cells from patients with autoimmune diseases (100).

In order to successfully treat autoimmune diseases and induce long-lasting tolerance, Treg dysfunction as well as the impaired T cell responsiveness towards immune regulation should be taken into consideration when establishing new therapeutic approaches. Common first-line therapies in the treatment of autoimmune diseases rely on a systemic immunomodulatory effect in the patient to rebalance the misguided immune system. In this context, it is interesting to investigate whether these disease-modifying drugs (DMD) also affect the regulation of the disturbed T cell population. Fortunately, we have been able to identify certain DMD with a positive effect on T cell susceptibility to Treg-mediated suppression. Treatment with dimethyl fumarate (DMF, Tecfidera) has been shown to completely reverse Treg resistance in CD4^+^CD25^-^ T cells isolated from the peripheral blood of RRMS patients (107). By co-culturing isolated CD4^+^CD25^-^ Teff from DMF-treated RRMS patients with functionally active Treg from healthy donors, we observed a pronounced suppression of T cell proliferation comparable to isolated Teff from healthy controls. This restored Treg sensitivity of T cells from DMF-treated RRMS patients correlates with normalization of IL-6R expression. In addition to DMF, treatment with IFN-β (Rebif) showed similar effects. *Ex vivo* analyses of isolated Teff from the peripheral blood of therapy-naïve versus IFN-β-treated RRMS patients showed that T cell responsiveness to Treg-mediated suppression is restored after IFN-β therapy (108). Furthermore, IL-6R expression as well as IL-6 production in T cells from IFN-β-treated RRMS patients are markedly diminished in comparison to untreated patients.

Further studies need to investigate the effect of other DMD used in the treatment of MS. Additionally, these investigations should be extended to other autoimmune diseases, such as RA or SLE, which are also characterized by a reduced T cell susceptibility to Treg-mediated suppression.

## Immunomodulatory drugs and Treg function

Since it has been reported that, in a variety of autoimmune diseases, Treg frequencies are reduced and/or the function of Treg is disturbed, this cell population has become of great interest for therapeutic intervention. In this part of the review, we discuss immunomodulatory drugs, used in the treatment of MS and RA, and their effect on Treg frequency and function. All findings are summarized in **Table 2**.

**Table 2:** Effect of immunomodulatory drugs on Treg frequency and function in MS and RA patients.

Autoimmune disease	Treatment	Treg in peripheral blood	Effect onTreg frequency	Effect on Treg function	Study
MS	Glatiramer acetate (Copaxone)	CD4^+^CD25^+^Foxp3^+^(total Treg) CD4^+^CD25^+^Foxp3^+^ CD45RA^+^CD45RO^-^(naïve Treg)CD4^+^CD25^+^Foxp3^+^ CD45RA^−^CD45RO^+^ (memory Treg) CD4^+^CD25^+^Foxp3^+^CD45RA^+^CD45RO^-^CD31^+^ (RTE-Treg)	Increased Increased No effect Reduced	Enhanced suppressive function	Haas et al. (109)
MS	IFN-β-1a (Rebif, Avonex)	CD4^+^CD25^+^FoxP3^+^ (total Treg) CD4^+^CD25^+^FoxP3^+^ CD45RA^+^CD45RO^−^ (naïve Treg) CD4^+^CD25^+^Foxp3^+^ CD45RA^−^CD45RO^+^ (memory Treg) CD4^+^CD45RA^+^ CD45RO^−^CD31^+^ Foxp3^+^ (RTE-Treg)	No effect Increased Reduced Increased	Enhanced suppressive function	Korporal et al. (110) De Andrés et al. (111)
MS	Dimethyl fumarate (Tecfidera)	CD4^+^CD127^low^ CD25^high^Foxp3^+^ Helios^−^	Increased	No data	Gross et al. (112)
RA	anti-IL-6R mAb (Tocilizumab)	CD4^+^CD25^high^Foxp3^+^ (total Treg)	Increased	No data	Kikuchi et al. (113)
RA	anti-TNF-α mAb (Etanercept)	CD4^+^CD25^high^Foxp3^+^ (total Treg)	Increased	No data	Huang et al. (114)

This table provides an overview of common immunomodulatory drugs and their effect on the frequency of individual Treg subpopulations in the peripheral blood of MS and RA patients. In addition, the influence of the DMD on the Treg function is listed. “No data” means that, according to the current state of research, no scientific studies are available showing an effect of the respective DMD on Treg function.

Up to now, there are some drugs reported that have beneficial effects on Treg in RRMS patients. Glatiramer acetate, the polymer of glutamic acid, lysine, alanine, and tyrosine, showed a rapidly changed Treg frequency after administration whereby especially the naïve Treg frequency was increased. The exact mechanism behind the enhancement in Treg frequency remains unclear, but it is speculated that there might be an immunomodulatory effect on thymic T cell development. In addition to the increased frequency, the immunosuppressive function of Treg was also restored (109).

Treatment with IFN-β, which has been used in clinics for almost 30 years, does not affect the overall Treg frequency in the peripheral blood of MS patients. Interestingly, a shift from a memory Treg phenotype towards a naïve Treg phenotype can be detected. A slight improvement in the suppressive function of Treg can be observed as early as four weeks after IFN-β administration. This improvement peaks six months after treatment: At this point, the suppressive function of Treg in MS patients is fully restored and comparable to the Treg function in the peripheral blood of healthy donors (110, 111). However, the exact mechanism of how IFN-β treatment enhances Treg function needs to be further investigated.

DMF is another drug used to modulate the misguided immune system in RRMS patients. Studies by Gross et al. showed that DMF therapy positively affects the total Treg number in the peripheral blood of MS patients (112). DMF promotes Treg development under Th17-polarizing conditions, which was linked to DMF-induced inactivation of GAPDH (115). Further *in vitro* studies by Ghadiri et al. revealed a decreased susceptibility of Treg to DMF-induced apoptosis compared to conventional T cells (116). Unfortunately, to date there are no results available which indicate a potential effect of DMF therapy on the suppressive function of Treg.

Some immunomodulatory drugs used in the treatment of RA patients also have an increased impact on Treg frequency and function. Pro-inflammatory cytokines such as IL-6 and tumor necrosis factor-α (TNF-α) play a crucial role in the pathogenesis of RA. The blockade of IL-6-mediated signaling by Tocilizumab, a humanized IgG1 monoclonal antibody (mAb) (117), results in a significant enhancement of total Treg numbers in the peripheral blood of RA patients and correlates with disease remission (113). The underlying mechanism has not yet been fully elucidated. However, studies by Dominitzki et al. showed that IL-6 has a negative effect on Treg development and function due to the inhibition of Foxp3 expression (118). These findings provide a possible explanation for the change in Treg frequency in RA patients treated with Tocilizumab. Based on the data available to date, it can be postulated that Tocilizumab might also restore the suppressive function of Treg. Studies by Samson et al. revealed a strong increase in the ability of Treg to suppress Teff proliferation in patients with giant cell arteritis undergoing Tocilizumab treatment (119).

Another successful therapeutic approach for RA patients is the blocking of TNF-α activity with anti-TNF-α mAb (120). Patients who received anti-TNF-α therapy show an enhancement of the overall Treg frequency as well as a pronounced suppression of Teff. Moreover, the increased Treg frequency was accompanied by RA disease remission (114). Previous data show that TNF-α induces apoptosis in Treg and decreases their suppressive function due to downregulation of Foxp3, which would explain the changes in Treg numbers after anti-TNF-α mAb administration (121–123).

Overall, there is a wide range of drugs in the treatment of autoimmune diseases, in particular MS and RA, which lead to an increase in Treg function and/or improvement of the suppressive capacity of Treg *via* different mechanisms and target structures. Unfortunately, some of these drugs lacking data in which the immunomodulatory effect on Treg is directly linked to disease activity

## Treg-based therapies of autoimmune diseases

Physiological Treg immunosuppression is induced upon specific TCR and co-receptor stimulation and is the most important mechanism of self-tolerance and immune regulation. However, Treg immunosuppression is impeded or blocked *in vivo* by a strong inflammatory environment and T effector cell resistance, too small numbers of Treg, genetic defects, or other unknown effects. Subsequently, impaired or hindered immunosuppression is an important trigger for unleashed autoimmunity, immunodeficiency disorders or other unwanted immune reactions such as graft-versus-host disease (GvHD), hypersensitivity reactions and allergies. Intriguingly, it has been shown recently that Treg effectively can exert their immunosuppressive activity in an established inflammation and strong inflammatory environment as long as Foxp3 expression is not lost (124). Excitingly, this finding may offer new opportunities for future Treg-based therapies.

Preclinical studies using animal models have provided first evidence for the concept of Treg cell-based therapies to overcome a dysregulated immune response in autoimmune diseases. It has been shown that adoptively transferred Treg could reduce the pathology in several autoimmune disease-related mouse models. A comprehensive compilation is given by Baeten et al. (125). Further developed humanized immunodeficient mouse models provided the opportunity to analyze human Treg function (126). These studies built the basis for the clinical translation of therapeutic Treg as a powerful alternative to immunosuppressants for treating autoimmune diseases. Today, the potential of Treg cell-based and non cell-based therapies as a therapeutical approach for autoimmune and other T cell-driven diseases is widely recognized. Treg therapies offer the opportunity to substitute life-long and high-dosed immunosuppressive medication without long-term interference with immune regulation. Increased susceptibility to opportunistic infections and malignancies as seen with systemic immunosuppression is not anticipated. Positively, this assumption could be confirmed in clinical trials so far (127). Due to the physiological mechanism of suppression, Treg therapies are even expected to have the potential to induce tolerance and cure autoimmune diseases and other T cell-driven pathologies.

### Polyclonal Treg as the primary basis of Treg cell-based therapies

In first clinical studies using Treg to treat autoimmune diseases, polyclonal Treg were applied in patients with T1D, showing modest but promising results and importantly, being safe (128, 129). However, the proof-of-concept was delivered leading to a steadily growing number of clinical trials with different and improved modalities in a variety of clinical settings. A comprehensive compilation of clinical studies using Treg is given by Baeten et al. (125). The most challenging hurdle for successful adoptive Treg therapies is to provide or induce a clinically effective number of Treg exerting an efficacious suppressive capacity. Starting point of all approaches is the isolation of Treg from leukapheresis or umbilical cord blood samples. Unfortunately, the number of Treg that can be isolated from these sources is limited making expansion and/or activation or genetical modification necessary prior to cell therapy.

### Functional requirements and cellular properties important for an efficacious Treg-based cell therapy

Cell-based strategies enabling Treg to be clinically efficacious, focus on the source of Treg, number of provided Treg, conditioning of Treg, applying combination therapies and the immunosuppressive potency of Treg.

A variety of known drugs and biologicals were identified to modulate Treg physiology and act as Treg growth factor, e.g. low dose IL-2, Treg-stabilizing factors, e.g. rapamycin and retinoic acid (127) or might enhance the activation of Treg, e.g. anti-CD3 and CD28 mAb which are also a basic trigger for Treg expansion. The mechanism of action of these mediators can be direct or indirect. Rapamycin, which induces Treg and phenotype stability directly, also inhibits non-Treg proliferation thereby backing indirectly the Treg population *in vivo* and *in vitro* (130). The JAK1/2 kinase inhibitor ruxolitinib favors the ratio of Treg vs. conventional T cells and seems also to enhance the suppressive capacity of Treg (131). Low-dose IL-2 and rapamycin are important factors for the *in vitro* manufacturing of Treg as well as in Treg cell therapies to complement and support adoptively transferred Treg. Interestingly, low-dose IL-2, given as a monotherapy in a clinical study to assess the Treg expanding and activating potential in a variety of autoimmune diseases gave hints for a potential clinical efficacy (132). Moreover, modified IL-2 biologicals exerting a longer half-life and improved specificity for Treg are being developed (133). The anti-CD20 mAb rituximab was shown in a combination therapy with Treg to be superior to a Treg monotherapy in a T1D clinical study (134).

However, most of these modulators are not Treg specific as they also hit conventional T cells and other cellular contaminates thereby generating unwanted activities and phenotypes in Treg expansion cultures. Furthermore, it was shown that Treg may lose their functional phenotype and lineage upon prolonged expansion making re-isolation of stable Treg from these heterogenous cultures difficult (135). These cellular impurities and unstable Treg represent a serious safety risk and a quality problem for the adoptive transfer of *in vitro* expanded polyclonal Treg.

### Polyclonal vs. antigen specific Treg cell therapy

For historical and pragmatic reasons most clinical studies applying Treg to suppress autoimmunity or to treat GvHD currently use polyclonal Treg. Polyclonal Treg manufactured in expansion cultures can be provided in sufficient numbers and exert suppression through both antigen-specific and unspecific bystander mechanisms. However, in follow-up studies with T1D it was shown that the efficacy of first applied polyclonal Treg cell therapies is limited and inferior to approaches applying antigen specific Treg. These cells can be generated by expansion with APC and the respective autoantigen or provided by genetically modified recombinant TCR Treg. It could be shown that a lower number of antigen specific Treg display a superior efficacy than polyclonal Treg in a transgenic NOD mice model (136). Apparently, antigen specific Treg have a higher capability and better homing ability. Polyclonal Treg preparations harbor multiple TCR specificities whereof only a minor number of Treg will be activated in an antigen-specific manner and exert suppression largely by antigen-independent bystander suppression. However, application of antigen specific Treg is limited to autoimmune settings with known and limited number of autoantigens whereas polyclonal Treg may be appropriate for systemic diseases with unknown autoantigens.

An interesting opportunity for polyclonal Treg cell therapy is the use of third-party allogeneic Treg instead autologous patient derived Treg. As Treg will not induce allogenic responses in the recipient, this approach allows HLA-independent ‘off-the-shelf’’ Treg supply for clinical application. This holds not true for recombinant TCR-expressing Treg, which are strictly MHC dependent and do not work in MHC-mismatched settings which limits their clinical application. This drawback has been overcome by the technology for the generation of chimeric antigen receptor Treg.

### CAR-Treg cell therapy

Chimeric antigen receptor Treg (CAR-Treg) represent a further development of CAR T cell technology generated for cancer therapies and represent engineered redirected Treg with artificial TCR, designated CAR. These artificial T cell receptors are composed of an extracellular domain for antigen recognition and binding and intracellular CD3 and CD28 domains for Treg activation upon antigen binding without MHC restriction. The MHC independent specific antigen recognition and subsequent functional activation enables a widespread application in patients as allogeneic Treg. Additional advantages over conventional Treg from expansion cultures seems to be a stable Treg phenotype and a more potent and specific immunosuppression. However, appropriate CAR specificities must be identified and engineered. So far, no clinical data are available with CAR-Treg. The first preclinical data with CAR-Treg were generated in mouse models of colitis and in a humanized mouse transplantation model. CAR-Treg exerted bystander suppression and could improve colitis and alloantigen-specific suppression (137). Important to mention are safety concerns in CAR-Treg therapies regarding potential cytotoxicities and a Treg-mediated target killing. Manufacturing of CAR-Treg is much more complex and challenging compared to other Treg cell therapies. Ongoing studies will give more information on the therapeutic potential of CAR-Treg cell therapies.

## Treg-specific activators

Besides the *in vitro* enabling technologies providing antigen-specific TCR and artificial CAR constructs or generating expanded polyclonal or antigen-specific Treg, there is another viable option to generate suppression-competent Treg. It was shown that the application of Treg-specific activators like GARP (glycoprotein A repetitions predominant or LRRC32) or gp120 represents a promising therapeutic approach.

### GARP

GARP is expressed on the surface of Treg and is the receptor for Latent-TGFβ1. Stimulation of Treg leads to an upregulation of GARP expression and subsequent release of soluble GARP (sGARP)/Latent-TGFβ1 complexes (138). By applying a recombinant sGARP (without Latent-TGFβ1) *in vitro* it could be shown that sGARP induces Foxp3 and shifts the differentiation of naive CD4^+^ T cells into induced Treg. In a preclinical humanized mouse model of xenogeneic GvHD, sGARP significantly enhanced Treg activity resulting in a suppression of lethal GvHD (139). But so far, no functional data were generated in autoimmune settings or in adoptive Treg cell therapies. As sGARP has immunoregulatory activities it may have a role in Treg manufacturing and Treg cell-based intervention in autoimmunity. Interestingly, a recently identified unique subset of tissue-resident γREG^+^ Treg is characterized by an increased expression of GARP and suggested to contribute to the long-term persistence of Treg (124).

### HIV gp120

In early preclinical studies anti-CD4 mAb have been successfully analyzed to induce immune tolerance in a variety of animal models. The goal was to eliminate or block CD4^+^ Teff rather than induction and activation of Treg. Although the translation of the anti-CD4 concept into the clinic was not successful, e.g. in RA patients (140) the concept of anti-CD4 tolerance induction was further developed and finally could be linked to human Treg induction and activation (141, 142).

**Figure 2:**
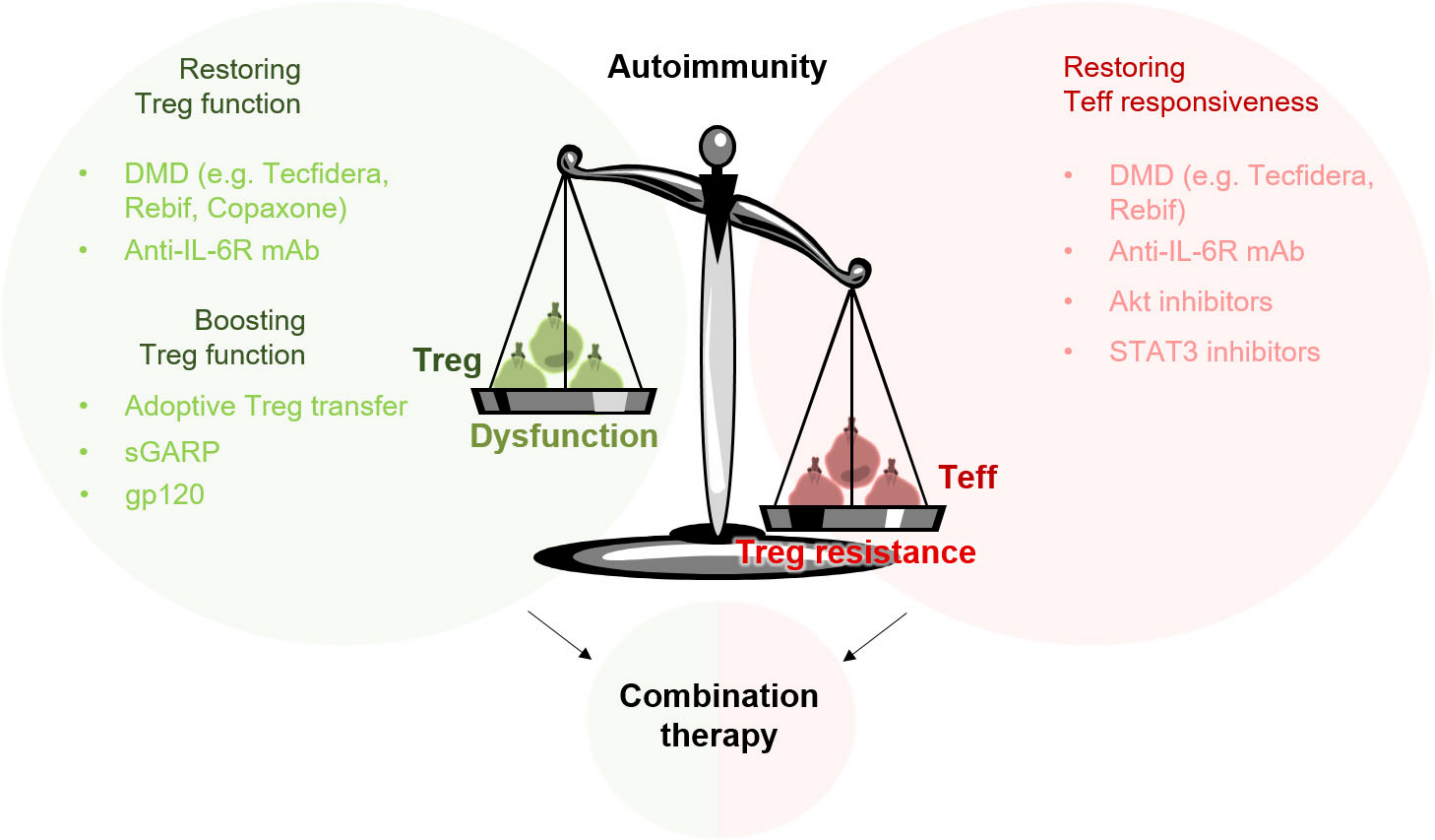
Targeting Treg resistance and Treg dysfunction in the treatment of autoimmune diseases. The imbalance of the immune system in patients with autoimmune diseases can be attributed to a disturbed function of Treg (Treg dysfunction) and to a reduced sensitivity of Teff to Treg-mediated suppression (Treg resistance). To rebalance the misguided immune system, a combination therapy targeting both sides – Treg dysfunction and Treg resistance – could be beneficial. Adoptive Treg transfer or Treg-specific activators such as gp120 can provide an additional boosting of the suppressive properties of Treg.

Intriguingly, the human immunodeficiency virus-1 (HIV-1) envelope glycoprotein gp120, which is the primary HIV receptor for human CD4 was shown to activate human Treg (143). Gp120-activated Treg prevent the development of lethal GvHD induced by adoptive transfer of human PBMC into immunodeficient mice and abrogated airway hyperresponsiveness in a humanized mouse model of allergic airway disease induced by PBMC from donors allergic to birch pollen (144). There are no data available from models for autoimmune diseases. Together, these findings demonstrate that stimulation *via* the human CD4 receptor represents an alternative Treg activating pathway with the potential to induce immunologic tolerance *in vivo* not restricted to GvHD (145). The discussed approaches to boost the Treg function are depicted in **Figure 2**.

## Preclinical testing of Treg-activating substances

Modulating the suppressive function of Treg represents an attractive therapeutic option for treating diseases associated with impaired Treg function. In autoimmune diseases such as MS or RA, which are characterized by a significantly reduced Treg function (3, 4, 146, 147), current strategies aim to restore Treg function by the use of adoptive Treg transfer or Treg-activating/-enhancing substances (127). But how can such substances be tested preclinically?

In general, a simple method to determine whether a substance activates Treg or not is the flow cytometric detection of proteins whose expression is altered by the activation stimulus. Up to now, a number of activation-induced surface markers have been described to specifically identify activated Treg after polyclonal or antigen-mediated stimulation and expansion *ex vivo*. Studies by Tran et al. revealed latency-associated peptide (LAP) and IL-1 receptor type I and II (CD121a/CD121b) as two specific surface markers that distinguish activated Foxp3^+^ Treg from activated Foxp3^+^ non-Treg after *ex vivo* expansion (148). GARP represents another cell-surface molecule, which is selectively expressed in Treg and up-regulated after TCR-mediated activation with anti-CD3/CD28 and IL-2 (149). In addition, studies by Schoenbrunn et al. showed that activated Treg differ from activated conventional T cells in their expression of 4-1BB (CD137) and CD40L (CD154). After polyclonal stimulation with PMA/ionomycin or allogeneic APC, activated Treg are defined by expression of CD137 and lack of CD154 (150). This CD137 and CD154 expression signature can be further used to specifically identify Treg even after prolonged *in vitro* expansion culture. Nowak et al. demonstrated that expanded Treg can be clearly distinguished from activated conventional T cells by the presence of CD137 and the absence of CD154 (135). However, the expression of these markers identified so far mainly relates to Treg that were expanded in a time-consuming *in vitro* culture using polyclonal activation. To what extent these markers can be transferred to activated, non-expanded Treg, e.g. gp120-activated Treg, needs to be further analysed. All in all, finding a universal signature for activated Treg is of great interest, as it would significantly speed up the functional testing of Treg potency *in vitro* for clinical application.

In addition to the flow cytometric analysis of activation-induced surface molecules, the efficiency of Treg-activating substances can be tested in an *in vitro* suppression assay. Although this analysis method is significantly more complex and time-consuming than flow cytometric analysis, the result provides direct information about the suppressive capacity of activated Treg. In the context of gp120-mediated activation of Treg, Becker et al. describe a useful *in vitro* coculture assay for testing the potency of stimulated Treg (143). Here, resting CD3^+^CD4^+^CD25^+^ Treg are cocultured in the presence or absence of increasing amounts of gp120 with syngeneic T-cell-depleted PBMC (as costimulus) and allogeneic CD8^+^ effector T cells. Cultures that are polyclonally stimulated with anti-CD3 mAb serve as a positive control. Proliferation of alloreactive CD8^+^ Teff is determined after three days of culture by the incorporation of radioactive thymidine. The results show that in this allogeneic setting, resting Treg are not able to efficiently suppress the proliferation of effector T cells. However, in the presence of gp120, stimulated Treg significantly prevent activation and proliferation of allogeneic CD8^+^ Teff in a dose-dependent manner. With the help of this assay, the efficiency of Treg-activating substances can be clearly examined. A disadvantage of this method, however, is that the setting and results are highly dependent on the alloreactivity of the immune cells. If the alloreactivity of both donors is less pronounced, the result cannot be clearly interpreted.

In order to overcome the limitations of *in vitro* assays, a broad range of humanized mouse models for the preclinical investigation of various therapeutic approaches have been established over the past decades. The engraftment of immunodeficient mice with functional human immune cells and tissues have become increasingly important to study human diseases, such as GvHD or autoimmunity, in a preclinical setting. Moreover, humanized mouse models offer the advantage of testing the effectiveness of a drug directly in a pathological context (151).

The xenogeneic GvHD model is one well-established mouse model to investigate the potency of Treg-stimulating substances. This model is based on the transfer of human peripheral immune cells into immunodeficient mice, such as NOD/*Scid*γc^-/-^ or Rag2^-/-^γc^-/-^. The transferred human T cells are activated by APC and differentiate into IFN-γ- and IL-17-producing Teff that migrate to certain organs such as the liver, skin, and intestines, where they initiate a strong inflammatory response. This causes serious damage to the liver (hepatitis), intestines (colitis), and skin (dermatitis) of mice. Furthermore, these mice are characterized by a pronounced weight loss (143). Just one week after the transfer of human immune cells, a strong infiltration of T cells into the liver and intestines of mice is observed (152). The limited number of intrinsic Treg within the transferred human immune cells cannot prevent GvHD development. Interestingly, the formation of GvHD can be completely suppressed by targeted activation of intrinsic Treg. In studies by Becker et al., a single application of gp120 induced a powerful activation of intrinsic Treg, which prevented the activation and proliferation of pathologic Teff (143).

Since the development of GvHD strictly depends on T-cell-mediated inflammatory responses, this mouse model is a suitable tool to study the regulation of Teff as well as the potency of Treg-specific substances to boost Treg function.

## Conclusions

In this review, we highlight two critical issues that should be taken into consideration when developing new therapies to treat autoimmune diseases. In general, several autoimmune diseases are characterized by an imbalance between immune suppression and immune activation. This imbalance can be attributed to two disorders, which are present in critical immune cell populations. In addition to a significantly reduced number and function of Treg, many autoimmune diseases also show reduced responsiveness of effector cells to Treg-mediated suppression (153). To efficiently treat patients with autoimmune disease and to avoid relapses, both sides – Treg resistance in Teff as well as Treg dysfunction – probably need to be targeted. Furthermore, in order to induce long-lasting tolerance in these patients, we consider a combination therapy to be extremely beneficial (**Figure 2**).

Efficient abolition of Treg resistance and restoration of Treg function has already been demonstrated for several DMD. However, to which extent these effects persist in patients has not been sufficiently investigated to date. Although many DMD lead to a significant improvement in the symptoms of the disease, relapses cannot be completely ruled out. In order to reduce the occurrence of relapses and thereby improve the patient’s quality of life, an additional “boosting” of Treg function, by adoptive Treg transfer or administration of Treg-specific activators such as gp120, could be beneficial.

In recent years, there have been impressive advances in the development of new therapeutic strategies for the treatment of autoimmune diseases. These novel strategies are based, for example, on the inhibition of signal transduction pathways, such as the JAK/STAT pathway, or blockade of overexpressed ion channels, such as Kv1.3. Since cytokines are a key driver in autoimmune diseases, targeting their associated signaling pathway by inhibiting Janus kinases (JAK) is an attractive approach (154). Baricitinib is one JAK inhibitor, which has been approved for the treatment of RA patients (155). Besides JAK inhibition, efficient blockade of Kv.1.3 ion channels is another promising approach. Effector memory T cells in patients suffering from autoimmune diseases, are characterized by an upregulated Kv1.3 channel (156). Dalazatide is the first Kv1.3 channel inhibitor used in human for the treatment of psoriasis (157). Both strategies, JAK inhibition as well as blockade of Kv1.3, significantly dampen inflammation thus improving the patient’s quality of life. However, even if these strategies achieve a significant improvement in disease symptoms, it remains unclear how long this effect will last and whether Treg resistance is affected and long-lasting self-tolerance is restored in these patients. It is important to note that the induction of self-tolerance appears to play a key role in curing autoimmunity. For this reason, we think that - in addition to addressing inflammation - addressing the suppressive side through the use of Treg-specific activators or adoptive Treg transfer, for example, is also very important.

All in all, the idea of targeting Treg resistance and Treg dysfunction in a combined approach is still relatively new and in its infancy. Initial investigations in preclinical, humanized mouse models are necessary in order to obtain data about the efficiency and tolerability of this therapy method in autoimmune diseases.

## Author contributions

All authors listed have made a substantial, direct, and intellectual contribution to the work, and approved it for publication.

## Conflict of interest

JS and BW are employees of ActiTrexx GmbH. FJS is co-founder and CKO of ActiTrexx GmbH and has patents and pending patents on gp120 stimulation of Treg.

The remaining author declares that the research was conducted in the absence of any commercial or financial relationships that could be construed as a potential conflict of interest.

## Publisher’s note

All claims expressed in this article are solely those of the authors and do not necessarily represent those of their affiliated organizations, or those of the publisher, the editors and the reviewers. Any product that may be evaluated in this article, or claim that may be made by its manufacturer, is not guaranteed or endorsed by the publisher.
